# Magnetotactic molecular architectures from self-assembly of β-peptide foldamers

**DOI:** 10.1038/ncomms9747

**Published:** 2015-10-29

**Authors:** Sunbum Kwon, Beom Jin Kim, Hyung-Kyu Lim, Kyungtae Kang, Sung Hyun Yoo, Jintaek Gong, Eunyoung Yoon, Juno Lee, Insung S. Choi, Hyungjun Kim, Hee-Seung Lee

**Affiliations:** 1 Molecular-Level Interface Research Center, Department of Chemistry, KAIST, Daejeon 305-701, Korea; 2 Center for Cell-Encapsulation Research, Department of Chemistry, KAIST, Daejeon 305-701, Korea; 3 Graduate School of Energy Environment Water Sustainability (EEWS), KAIST, Daejeon 305-701, Korea

## Abstract

The design of stimuli-responsive self-assembled molecular systems capable of undergoing mechanical work is one of the most important challenges in synthetic chemistry and materials science. Here we report that foldectures, that is, self-assembled molecular architectures of *β*-peptide foldamers, uniformly align with respect to an applied static magnetic field, and also show instantaneous orientational motion in a dynamic magnetic field. This response is explained by the amplified anisotropy of the diamagnetic susceptibilities as a result of the well-ordered molecular packing of the foldectures. In addition, the motions of foldectures at the microscale can be translated into magnetotactic behaviour at the macroscopic scale in a way reminiscent to that of magnetosomes in magnetotactic bacteria. This study will provide significant inspiration for designing the next generation of biocompatible peptide-based molecular machines with applications in biological systems.

## Abstract (web summary)

Controlling organic materials with magnetic fields in a dynamic fashion is a challenging task. Here, the authors show that synthetic ß-peptide foldamers can be rotated at will under a dynamic magnetic field and that this can be extended to macroscopic scale objects containing these materials.

## Graphical abstract

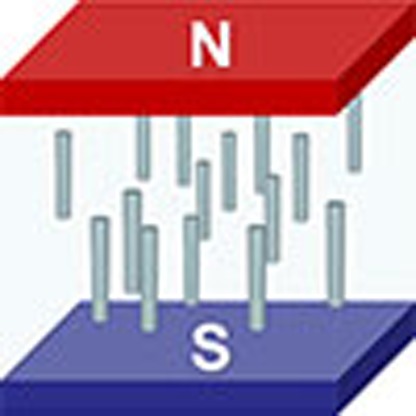

Foldamers have attracted much attention in synthetic and biological chemistry as molecular frameworks that can mimic the folding and organizing behaviours of biomacromolecules^1^,^2^,^3^. In recent years, considerable progress has been made in developing biotic and abiotic foldamers with diverse secondary structures, focusing mainly on biological and medicinal applications^4^,^5^,^6^,^7^. The next important challenge in this field is to design more complicated, higher-order structures by self-assembly of foldamer-building units, and thus to elicit desirable functions such as enzymatic catalysis and molecular recognition from the well-organized structures^8^,^9^,^10^,^11^, analogous to those of natural proteins. It has been reported that a wide range of foldamers can self-assemble into higher-order structures, including helical bundles, multiple helices, nanofibres, nanosheets, nanovesicles and liquid crystals^12^,^13^,^14^,^15^.

Recently, we reported unprecedented three-dimensional molecular architectures, named ‘foldectures', derived from self-assembly of β-peptide foldamers consisting of *trans*-(*S*,*S*)-2-aminocyclopentanecarboxylic acids (*trans*-ACPC)^16^,^17^,^18^,^19^. *Trans*-ACPC oligomers have exceptional helical stability and predictable intermolecular interactions^20^,^21^, and can therefore self-assemble under aqueous conditions to form unique anisotropic microstructures with controllable shapes, sizes and crystallinities. More importantly, foldectures display distinctly defined monodisperse morphologies, unlike conventional self-assemblies of natural peptides. We took advantage of their size uniformity and well-ordered molecular packing to use individual foldectures as stimuli-responsive, functional supramolecular architectures.

Here we show that foldectures can undergo real-time mechanical motions in response to an external dynamic magnetic field, and provide the detailed elucidation of the magnetic orientation of foldectures as well. This proof-of-concept experiment regarding peptide-based supramolecular machine demonstrates the capability of facile magnetic manipulation of diamagnetic organic materials in solution, which may offer a basic molecular system demanding for the development of potential biocompatible nano- and micromachines^22^.

## Results

### Alignment of foldectures by static magnetic field

The effect of diamagnetism at the single-molecular level is negligible compared with the thermal energy that results in random Brownian motion of molecules, even under strong magnetic fields of a few tesla. However, when diamagnetically anisotropic molecules are in a structurally well-ordered arrangement (for example, molecular crystals, crystalline polymers and carbon materials), the total diamagnetic anisotropy of the molecular system is assumed to be the sum of the anisotropic contributions from the individual constituents. The collective anisotropic magnetic energy, which is sufficient to overcome the thermal energy, generates mechanical torques to align the molecules in the most favourable orientations, with minimum energy states^23^,^24^,^25^,^26^,^27^,^28^,^29^,^30^,^31^,^32^,^33^,^34^,^35^. In this respect, foldectures are expected to be suitable molecular platforms for amplifying diamagnetic anisotropy at the molecular level and observing their macroscopic motions, because they have highly crystalline molecular packing and monodisperse morphologies at the microscale.

**Figure 1:**
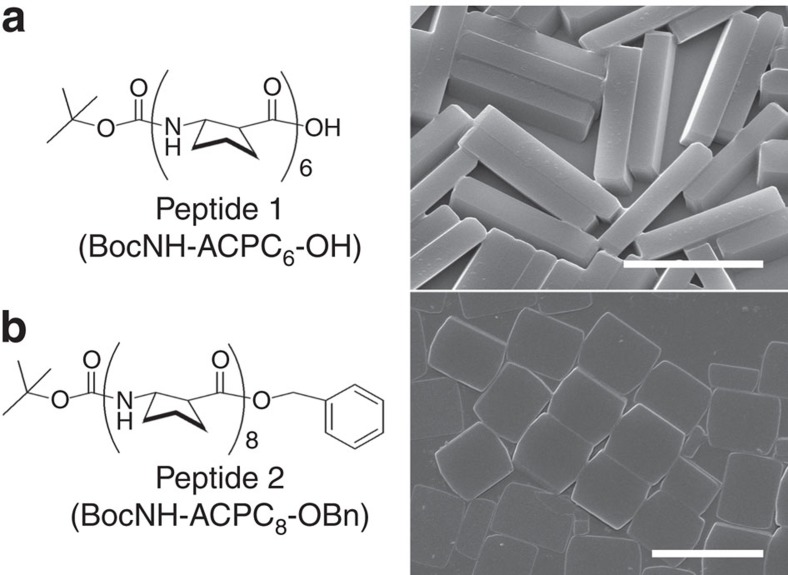
β-Peptide building blocks and SEM images of corresponding foldectures. (**a**) Peptide **1** (BocNH-ACPC_6_-OH) and SEM image of rhombic rod foldectures (**F1**). (**b**) Peptide **2** (BocNH-ACPC_8_-OBn) and SEM image of rectangular plate foldectures (**F2**). Scale bars, 5 μm. Boc, *tert*-butyloxycarbonyl.

**Figure 2:**
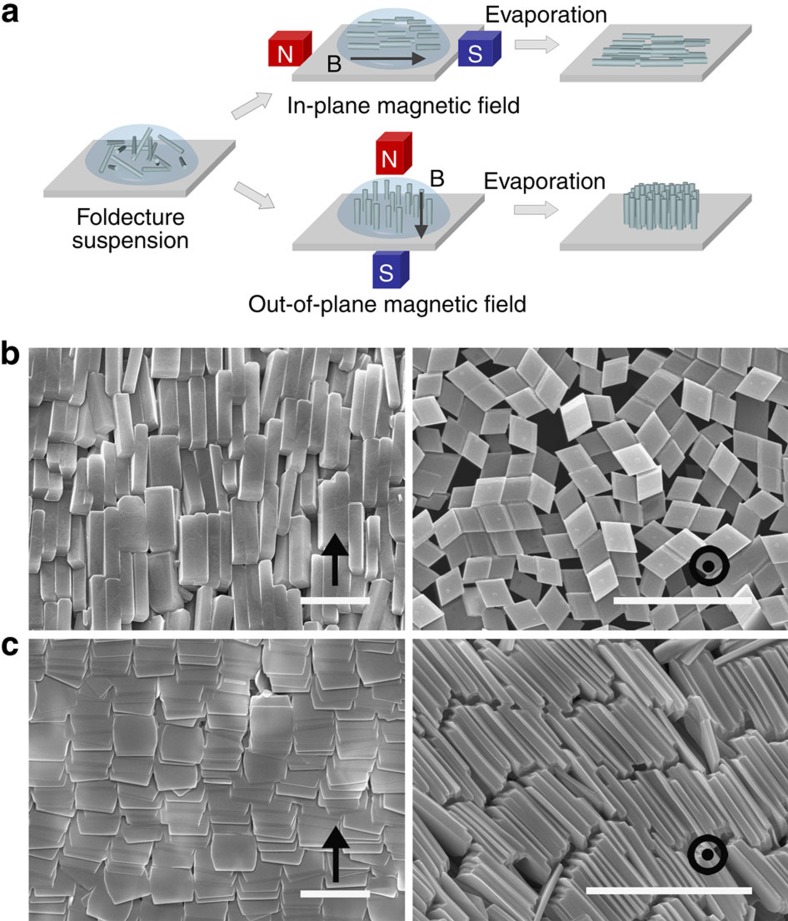
Alignment of foldectures by static magnetic field. (**a**) Schematic diagram of experimental process for alignment of foldectures under static magnetic field. (**b**) SEM images of rhombic rod foldectures (**F1**) deposited on Si substrates under (left) in-plane magnetic field and (right) out-of-plane magnetic field. (**c**) SEM images of rectangular plate foldectures (**F2**) deposited on Si substrates under (left) in-plane magnetic field and (right) out-of-plane magnetic field. Arrows and circles indicate direction of magnetic field. Scale bars, 5 μm.

We first investigated the effect of a magnetic field on two different types of foldecture: rhombic rods (**F1**) from peptide **1** (BocNH-ACPC_6_-OH, Fig. 1a) and rectangular plates with two rounded edges (**F2**) from peptide **2** (BocNH-ACPC_8_-OBn, Fig. 1b). Each foldecture was prepared by self-assembly of peptide **1** or **2** under aqueous conditions. An aliquot of an aqueous suspension of the foldectures was placed on a Si substrate, then allowed to dry in a strong magnetic field of 9.52 T under ambient conditions (Fig. 2a). Scanning electron microscopy (SEM) analysis showed that the foldectures aligned with respect to the external magnetic field to form a neatly packed array. In the case of rhombic rods **F1**, the rods aligned with their longitudinal axes parallel to the direction of the horizontal magnetic field (in-plane to the substrate; Fig. 2b, left). Under a vertical (that is, out-of-plane) magnetic field, the rods stood vertically on end, with their rhombic sides facing up (Fig. 2b, right). In contrast, in the case of rectangular plates **F2**, the minor axis of the rectangular face aligned parallel to the field direction; the rectangular plates were stacked neatly, with their rectangular faces facing up under a horizontal magnetic field (Fig. 2c, left). Whereas, a vertical magnetic field aligned the plates perpendicular to the substrate, such that a pile of plates stood vertically on the surface (Fig. 2c, right). It is worth noting that foldectures can align in geometrically unfavourable orientations on application of an external magnetic field.

### Origin of magnetic alignment

The magnetic alignment of the foldectures can be explained by collective diamagnetic anisotropy in their ordered molecular packing. The anisotropic magnetic energy for a diamagnetic crystal in an external magnetic field *H* is given by


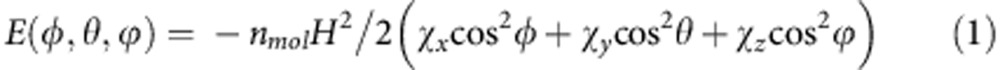


where *n*_*mol*_ is the mole number of molecules in the crystal, χ_*x*_, χ_*y*_ and χ_*z*_ are the molar diamagnetic susceptibilities along the principal magnetic *x, y* and *z* axes, and *ϕ*, *θ* and *ϕ* are the angles between the principal magnetic axes and the magnetic field direction. As described by Fujiwara *et al*.^28^, assuming that the anisotropic order is χ_*x*_<χ_*y*_<χ_*z*_<0, the anisotropic magnetic energy *E*(*ϕ*,*θ*,*ϕ*) is minimum when *ϕ*, *θ*=*π*/2, *ϕ*=0. The principal *z* axis, the largest (that is, the smallest absolute value) magnetic susceptibility axis, becomes the easy magnetization axis and tends to orient parallel to the applied field direction. On the basis of this theoretical background, determination of the anisotropic order of diamagnetic susceptibilities is essential for explaining the direction of foldecture alignment observed in experiments.

**Figure 3:**
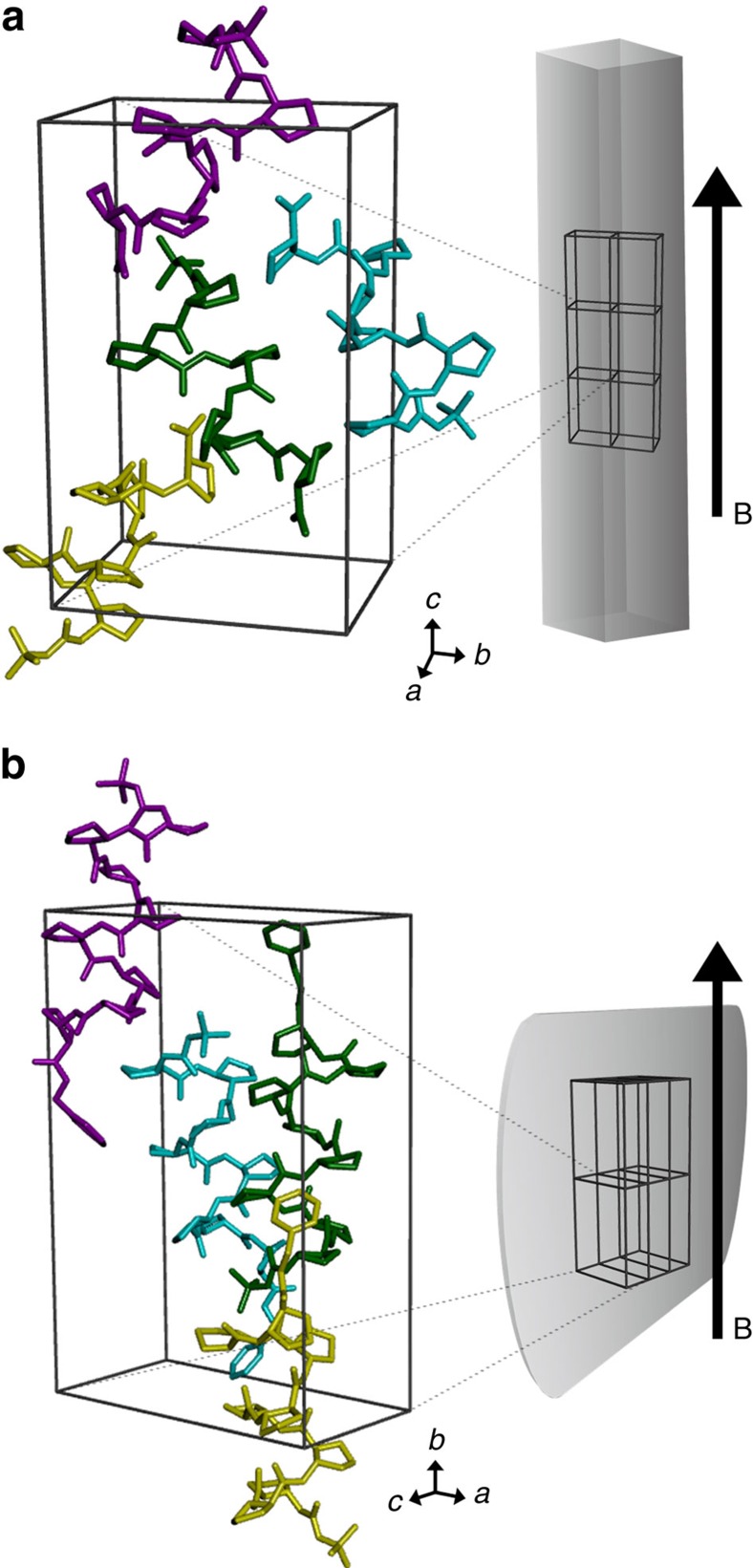
Molecular packing structures of foldectures in the unit cell and the direction of magnetic alignment. (**a**,**b**) Crystal packing arrangement of (**a**) rhombic rod foldecture **F1** and (**b**) rectangular plate foldecture **F2**. The four individual molecules in the orthorhombic unit cells are shown in magenta, azure, green and yellow. The illustrated orientations of the foldectures with respect to the magnetic field **B** represent the observed direction of alignment.

We calculated the diamagnetic susceptibilities of the crystal structures of foldectures **F1** and **F2**. Molecular packing in the foldectures was investigated using powder X-ray diffraction combined with single-crystal and electron diffraction experiments (Supplementary Fig. 3)^19^,^21^. The diamagnetic susceptibilities along the three orthogonal crystallographic *a*, *b* and *c* axes of the orthorhombic lattices were calculated using density functional theory (DFT). Owing to the practical limitations in computational time, where direct DFT calculation of four peptides in a unit cell is unavailable, the magnetic susceptibility tensor (*χ*_*ij*_) of each peptide molecule in a unit cell was individually calculated, and then their sum was calculated as the total susceptibility tensor (Supplementary Fig. 4). Here the *χ*_*ij*_ represents the magnetic susceptibility tensor component in the *i*th direction from the magnetic field applied in the *j*th direction. The contributions of the diagonal tensors in each peptide were combined to yield the total susceptibilities along the crystallographic axes, and the rest of the tensors cancelled out. In the case of **F1**, the diamagnetic susceptibility along the *c* axis, which corresponds to the longitudinal axis of the rod, had the largest (least negative) calculated value, −1837.7 × 10^−6^ cm^3^ mol^−1^ (Fig. 3a and Table 1). In the case of **F2**, the *b* axis, lying along the minor axis of the rectangular plate, was estimated to be the largest diamagnetic susceptibility axis (−2640.1 × 10^−6^ cm^3^ mol^−1^) (Fig. 3b and Table 1). These theoretically estimated susceptibilities well explain the magnetic alignment direction of foldectures observed in experiments. The foldectures showed the alignment of a single axis parallel to the applied static magnetic field, and the rest of the axes were not spatially fixed, displaying random distributions, as shown in Fig. 2b,c (ref. 29).

Generally, large diamagnetic anisotropy of organic molecules is attributed to delocalized electrons in the resonance structure, making the axis normal to the resonance plane more diamagnetic^36^,^37^. In our molecular scaffolds, the primary contribution to the diamagnetic anisotropy is expected to be from amide groups and benzene rings, which have resonance structures. Previous studies showed that oligomers of β-amino acids constrained by cyclopentyl rings fold preferentially in particular helical conformations via 12-membered hydrogen bonds, in which the orientations of the amide resonance planes align nearly parallel to the helical axis^6^. In the crystal structures of both **F1** and **F2**, helical columns of peptides are linked by head-to-tail intermolecular hydrogen bonds and laterally associated via an antiparallel neighbour relationship, that is, the average orientation of the helical axes is aligned parallel to the applied field direction (Supplementary Fig. 5). This interpretation is in good agreement with reports that many α-helical peptides tend to orient parallel to the applied field^33^. The benzene group on the C terminus of peptide **2** also seems to play a crucial role in diamagnetic anisotropy, therefore we calculated the diamagnetic susceptibilities for the main peptide body and benzene group separately. Peptide **2**, without a terminal benzene group, showed an easy magnetization axis along with its helical axis (*b* axis; Supplementary Fig. 6a). The orientations of the four benzene groups in the unit cell of **F2** increase the susceptibility along the *a* axis substantially, but this does not change the overall anisotropy order (that is, direction of alignment; Supplementary Fig. 6b).

**Figure 4:**
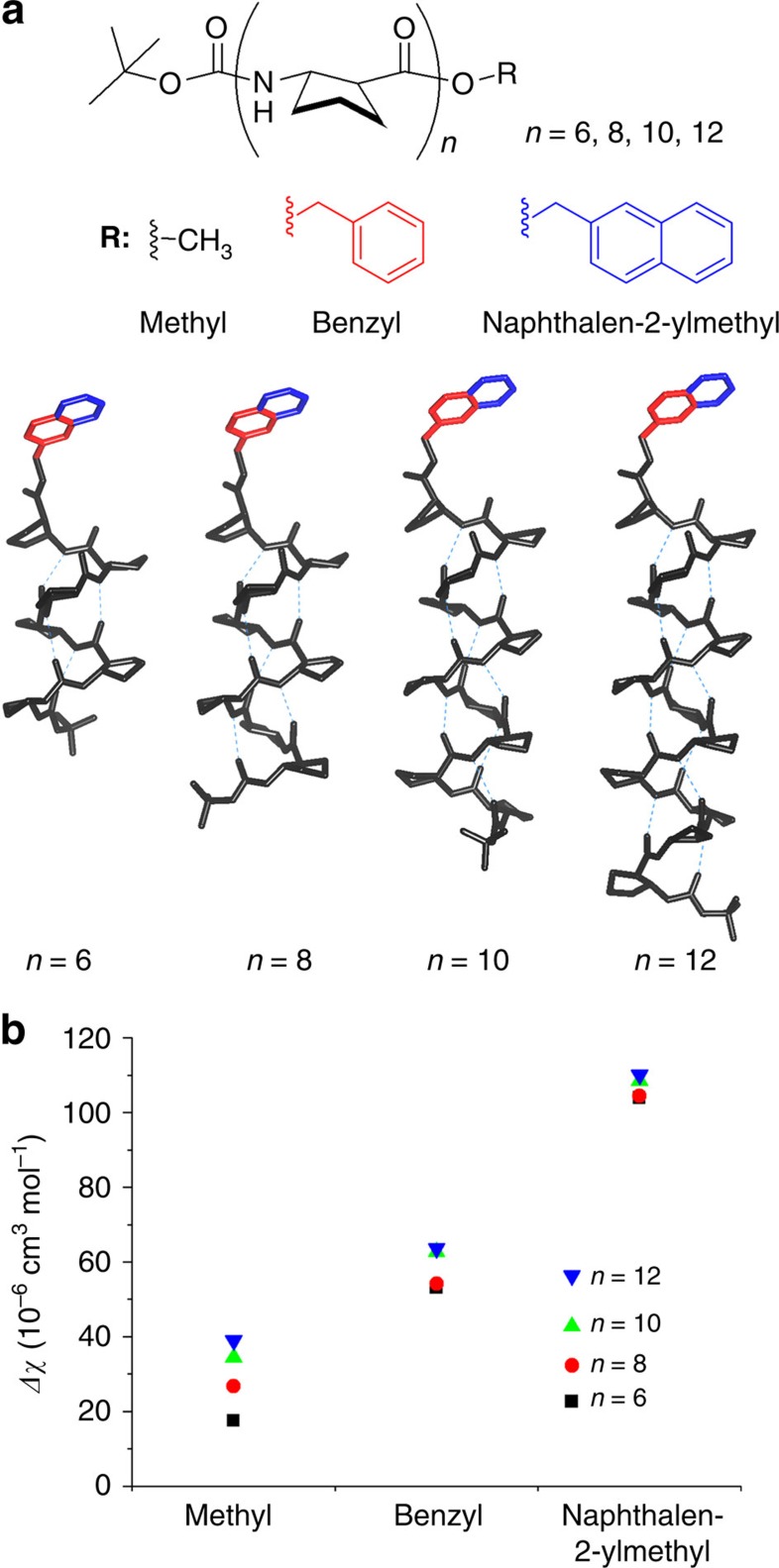
Diamagnetic anisotropy of model 12-helical peptides. (**a**) Model peptides (BocNH-ACPC_*n*_-OR, *n=*6, 8, 10 and 12) with various C-terminal functional groups (methyl, benzyl and naphthalene-2-ylmethyl) for DFT calculation of diamagnetic anisotropic susceptibilities. Sky-blue dashed lines indicate the 12-membered intramolecular hydrogen bonds. (**b**) Calculated maximum diamagnetic anisotropic susceptibilities (Δ*χ*) of the peptides (black squares: hexamers; red circles: octamers; green triangles: decamers; blue inverted triangles: dodecamers).

For more comprehensive understanding on the contribution of each functional group to diamagnetic anisotropy, and thereby to grasp an idea for molecular design of foldectures with maximized anisotropy, we examined the trend in diamagnetic anisotropy of model 12-helical peptides as a function of helical length and the type of C-terminal functional group. A series of *trans*-ACPC homooligomers with three different C-terminal functional groups (methyl, benzyl and naphthalene-2-ylmethyl) were investigated, in which the typical 12-helical conformation (that is, intramolecular hydrogen bonds between C=O (*i*) and N–H (*i*+3) of the backbone amides) and the spatial orientation of the C-terminal functional groups were fixed (Fig. 4a).

Using DFT calculations, maximum diamagnetic anisotropic susceptibility at molecular level for each peptide was estimated along with the direction of easy magnetization axis by diagonalizing the calculated magnetic susceptibility tensor. The estimated diamagnetic anisotropic susceptibilities for the peptides with methyl ester increased gradually as the helical length increased: the anisotropy for hexamer was 17.51 × 10^−6^ cm^3^ mol^−1^ (*n=*6, black square), which increased up to 38.99 × 10^−6^ cm^3^ mol^−1^ for dodecamer (*n=*12, blue inverted triangle; Fig. 4b). The average anisotropy per amide residue was estimated to be 3.24±0.3 × 10^−6^ cm^3^ mol^−1^. The result suggests that nearly parallel arrangement of the amide resonance planes along the helical axis allows the consistent contribution to the molecular anisotropy. On the other hand, introduction of functional groups having a large aromatic ring current resulted in significant effects on the molecular diamagnetic anisotropy. For example, the diamagnetic anisotropic susceptibilities of the peptides with aromatic esters, such as phenyl and naphthyl groups, were two to three times larger than those with the corresponding methyl esters (Fig. 4b). Meanwhile, the contribution from amide residues to the overall molecular diamagnetic anisotropy was accordingly attenuated by the presence of the aromatic functional groups. The differences in diamagnetic anisotropies between hexamers and dodecamers were 21.48 × 10^−6^, 10.55 × 10^−6^ and 6.15 × 10^−6^ cm^3^ mol^−1^ for the peptides with methyl, benzyl and naphthalene-2-ylmethyl esters, respectively. It is noteworthy that the directions of easy magnetization axes for the series of peptides varied considerably with helical length and the orientation of aromatic resonance plane as well (Supplementary Fig. 7). The introduction of higher diamagnetic groups seems to be positive for designing more sensitive diamagnetic molecules, but the net effect from the aromatic rings is apt to be offset by the spatial orientations in their assembled systems, as shown in Supplementary Fig. 6. Thus, more sensitive diamagnetic systems can be designed by appropriate combinations of higher diamagnetic molecules with their well-organized supramolecular packing modes, although it cannot be predicted a priori.

### Orientational motion of foldecture in dynamic magnetic field

**Figure 5:**
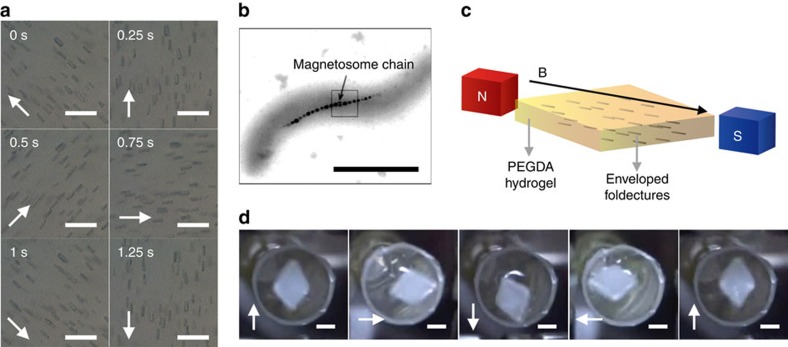
Real-time observation of magnetic orientation of **F1** and magnetotactic behaviour of macroscopic hydrogel container enveloping aligned **F1**. (**a**) Snapshots taken from optical microscopy videos showing foldectures **F1** suspended in water under rotating magnetic field. Arrows indicate direction of magnetic field. Scale bars, 10 μm. (**b**) Transmission electron microscopy image of a single cell of *Magnetospirillum magnetotacticum*^38^. Scale bar, 1 μm. (**c**) Schematic representation of magnetosome-inspired rhombus-shaped hydrogel container enveloping aligned foldectures **F1**. (**d**) Snapshots of macroscopic hydrogel container showing magnetotactic behaviour. Arrows indicate direction of magnetic field. Scale bars, 1 mm. Panel **b** is reproduced, with permission, from ref. 38 (copyright 2015, AAAS).

On the basis of these experimental results, we used foldectures directly as supramolecular architectures, which can show macroscopic movement when stimulated by an external magnetic field. We designed a simple experimental set-up that enables observation of real-time responses of foldectures to a dynamic magnetic field. Our laboratory-built rotating magnetic field device, consisting of a pair of 1-T permanent Nd–Fe–B magnets, was equipped with a high-resolution optical microscope (Supplementary Fig. 8). A horizontal rotating magnetic field was applied, and suspensions of foldectures on a glass substrate were directly monitored using this set-up. As the optical images show, while floating in a droplet, foldectures **F1** instantaneously aligned parallel to the direction of the applied field (Fig. 5a). On gentle rotation of the magnets, the foldectures displayed synchronized spinning motions to maintain the parallel orientation with respect to the rotating field (Supplementary Movie 1). In the case of **F2**, in-plane orientation of rectangular plates was identified: the minor axis of the rectangular plate oriented parallel to the field direction, whereas the major axis perpendicular to the field showed various orientations (Supplementary Fig. 9 and Supplementary Movie 2). The immediate orientational response of a foldecture can therefore be described as the trajectory of the dynamic magnetic field line. Collective anisotropy in the diamagnetic susceptibility gives rise to simultaneous orientation as the synchronized response to a subtle change in the dynamic field direction. These results suggest that the foldectures themselves can function as supramolecular machines, which can translate an external rotating magnetic field into spinning motion at the microscale.

### Magnetotactic foldectures as organic magnetosomes

A wide range of magnetotactic bacteria synthesize unique intracellular organelles, called magnetosomes, containing superparamagnetic nanocrystals as compass needles to perceive the direction of the Earth's magnetic field and passively align their bodies parallel to it (Fig. 5b)^38^. We envisioned that, in a similar manner, foldectures would be able to collectively transmit their rotational forces to a macroscopic object, by acting as magnetosome-inspired artificial organelles. We therefore devised a simple demonstration of macroscopic mechanical work stimulated by a dynamic magnetic field. To harness the microscale motions of foldectures as macroscopic movement, a large number of foldectures were incorporated into a macroscopic host object, and their orientations were tightly held by the hardening of the surrounding medium (Fig. 5c). First, highly concentrated rhombic rod foldectures **F1** were suspended in an aqueous solution of poly(ethylene glycol) diacrylate (PEGDA) with a trace amount of photoinitiator. The resulting mixture was poured into a rhombus-shaped polydimethylsiloxane (PDMS) mould of diameter 3 mm, and the foldectures were aligned using an external static magnetic field. While the magnetic field was applied, the mixture was exposed to ultraviolet light to induce photopolymerization to fix the foldecture orientations. After irradiation, a macroscopic, stiff hydrogel container enveloping the aligned foldectures was successfully obtained (Supplementary Fig. 10). The rhombus-shaped hydrogel container floated on the water, and instantaneously followed the rotation of the magnets, to indicate the direction of the magnetic field rotating at about 35 r.p.m. (Fig. 5d and Supplementary Movie 3). This macroscopic actuation is the result of hierarchical amplification of the diamagnetic anisotropy of a single peptide, implying that the enveloped foldectures can play a role similar to that of magnetosomes in magnetotactic bacteria. Although the strength of the magnetic field needed to align the foldectures is several orders of magnitude higher than that of the Earth's magnetic field, facile magnetic manipulation of diamagnetic materials has potential applications in biocompatible, remote-controllable micromachines operating in biological systems.

## Discussion

In summary, we showed that self-assembled molecular architectures of *β*-peptide foldamers (foldectures) align in response to an external magnetic field. The origin of the magnetic alignment was investigated using diamagnetic susceptibility calculations combined with structural analysis. Moreover, we demonstrated a magnetosome-inspired magnetotactic behaviour by implantation of magneto-responsive foldectures into the millimetre-sized hydrogel container. Owing to their well-defined morphologies and sufficient diamagnetic anisotropy, foldectures can function as supramolecular machines capable of translating dynamic magnetic field into instantaneous motions at the microscopic as well as macroscopic scales.

Light and heat have been often employed as external stimuli for conventional organic molecular machines performing macroscopic mechanical tasks, but magnetic fields have not been considered due to the small diamagnetic susceptibilities of organic materials^39^,^40^,^41^,^42^,^43^,^44^. As demonstrated in this study, magnetic stimulus can be a useful alternative for manipulating molecular machines. Furthermore, magnetic field allows sustainable operation because it does not essentially accompany the alteration of structural and chemical composition^25^.

Magnetically driven, non-invasive control of nano- and micro-objects in solution is a highly demanding technology for the development of versatile toolbox applicable to biological systems. In most of reported studies, nano- and micromotors made from paramagnetic and ferromagnetic materials have been used for biomedical applications (for example, drug delivery and targeted therapy)^45^,^46^,^47^,^48^. Although there have been considerable efforts to control the motions of diamagnetic materials especially using repulsive property^49^,^50^,^51^, the level of control is still in its infancy compared with those of paramagnetic and ferromagnetic materials. We hope that our results combined with technical improvement in modulating dynamic magnetic field in the future will offer attractive opportunities for the use of organic diamagnetic supramolecular materials as biofunctional nano- and micromachines.

## Methods

### Materials

All chemicals and solvents were purchased from commercial suppliers (Sigma Aldrich, Acros, Junsei, Novabiochem and TCI) and were used without further purification. High-purity water was generated by Milli-Q apparatus (Millipore).

### Preparation of peptides

Peptide **1** (BocNH-ACPC_6_-OH) and peptide **2** (BocNH-ACPC_8_-OBn) were prepared by solution phase peptide synthesis as described in the preceding literatures^21^,^52^.

### Self-assembly procedures for the production of foldectures

Rhombic rod **F1**: a solution of peptide **1** in tetrahydrofuran (200 μl, 2 g l^−1^) was injected into an aqueous solution of P123 (1 ml, 8 g l^−1^) with vigorous stirring for 3 min at room temperature. After an incubation of 3 h at the same temperature, the resulting white precipitate was isolated by centrifugation.

Rectangular plate **F2**: a solution of peptide **2** in tetrahydrofuran (200 μl, 1 g l^−1^) was injected into an aqueous solution of P123 (1 ml, 8 g l^−1^) with vigorous stirring for 3 min at room temperature. After an incubation of 3 h at the same temperature, the resulting white precipitate was isolated by centrifugation.

### Characterization

SEM images were obtained using a field-emission scanning electron microscope (Inspect F50, FEI, USA) at an accelerating voltage of 10.0 kV, after Pt coating (sputter coater 108auto, Cressington Scientific Instruments, UK). Transmission electron microscopy image and selected area electron diffraction patterns of the rectangular plate foldectures **F2** were obtained using a transmission electron microscope (Tecnai G2 30, FEI, USA) operated at 200 kV. Samples were deposited on a formvar carbon film on a 200-mesh copper grid (Electron Microscopy Sciences, USA). Optical microscopy movies were recorded using a three-dimensional digital microscope system equipped with a charge-coupled device camera (KH-8700, Hirox, Japan).

### Magnetic alignment of foldectures

Magnetic alignment of the foldectures in a vertical magnetic field of 9.52 T was performed in the superconducting magnet of a nuclear magnetic resonance spectrometer (AV400, Bruker, USA). An aqueous suspension of foldectures (3 μl) was placed on a Si (100) substrate and allowed to dry inside a vertical static magnetic field at the centre of the solenoid bore. For a horizontal magnetic field, the Si substrate was loaded inside the bore with 90° tilting. After complete evaporation, the samples were observed using a scanning electron microscope (Supplementary Figs 1 and 2).

Magnetic alignment in a horizontal rotating magnetic field was performed using the laboratory-built rotating magnetic field device. An aqueous suspension of foldectures (1 μl) was placed on a glass substrate, which was located between the central nodes of the magnets where the strongest field is generated. The samples were monitored in real-time using an optical microscope.

### Structure determination by powder diffraction pattern

The sample of rectangular plate foldectures **F2** was prepared for powder X-ray diffraction experiment as dried powder form. We carried out the experiment at the 9B HRPD beamline in Pohang Accelerator Laboratory (PAL, Pohang, Korea). Post-data processing for the obtained pattern was proceeded using the beamline's own programme (merging, normalization and peak asymmetry correction). *Crysfire*^53^ suite was used for indexing, and gave a unit cell of orthorhombic system with following dimension (*a=*20.488 Å, *b=*30.552 Å and *c=*10.208 Å) with high figure of merit (=37.39). To determine the space group for this sample, the programme *Chekcell*^54^ was modified for only Sohnke space groups. This procedure recommended *P*2_1_2_1_2_1_ or *P*2_1_2_1_2 as the appropriate space group for peptide **2**, and *P*2_1_2_1_2 space group was finally selected according to the *R* factor in the structure solution phase. *FOX*^55^ was used for the structure solution phase. In this step, many restraints were used to describe the complex foldamer's secondary structure and also its circumstances, such as anti-bump restraints, intramolecular hydrogen bonds, global Biso and so forth. March-Dollase preferred orientation parameter (100 direction) was refined during the solution step because of the plate shape of the foldecture. The obtained structure was used as the initial structure for the following Rietveld refinement. Using *GSAS/EXPGUI*^56^ package, the refinement step was done using typical method, as described in our previous report. The detail information obtained from this structure determination by powder diffraction pattern is described in Supplementary Fig. 3 and Supplementary Tables 1 and 2.

### Diamagnetic susceptibility calculations

First-principle DFT calculations of the diamagnetic susceptibility were performed using the Vienna *ab initio* simulation package (VASP 5.3; ref. 57). The generalized-gradient approximation of the Perdew–Burke–Ernzerhof^58^ exchange-correlation functional and projector-augmented wave type electron–ion interaction were used, with a plane wave energy cut-off value of 500 eV, and only gamma-point sampling in reciprocal space. The ground-state electronic structure of each peptide molecule in a unit cell was calculated using single-point calculation based on experimental crystal structures. Magnetic susceptibility tensor components were calculated using gauge-including projector-augmented wave method using the linear response method of Pickard and Mauri^59^,^60^. The minimum (threshold) magnetic field strength is obtained when the anisotropic magnetic energy of a crystal equals to the thermal energy. The equation is given by


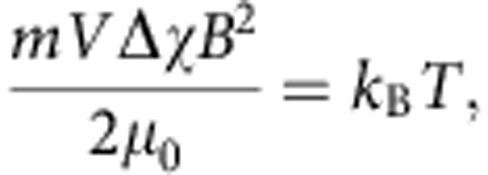


where *m* is the number of unit cells in crystal, *V* is the volume of a unit cell, Δ*χ* is the calculated anisotropic diamagnetic susceptibility of the unit cell, *B* is the applied magnetic field strength, *μ*_0_ is the magnetic permeability of vacuum, *k*_B_ is the Boltzmann constant and *T* is the temperature. Theoretically estimated minimum magnetic field required to align foldecture **1** (average volume of 1.04 μm^3^) is 0.21 T, which is in good agreement with the experimentally observed minimum magnetic field of 0.22 T. The estimated minimum magnetic field for foldecture **2** (average volume of 0.48 μm^3^) is 0.30 T, which is also in good agreement with the experimentally observed minimum magnetic field of 0.31 T.

### Design of laboratory-built rotating magnetic field device

Two attractive cylindrical Nd–Fe–B magnets (diameter 25 mm, height 45 mm, Magnetpark Inc., Korea) were brought together with an interspace of 7 mm, with plastic spacers, to generate a strong horizontal magnetic field between the magnets (Supplementary Fig. 8). The set of magnets was installed on a rotating wheel, which was manipulated manually using a roller. The maximum magnetic field strength at the centre of the device was determined to be 1.01 T, using a 475 DSP Gaussmeter (Lake Shore Cryotronics Inc., USA).

### Fabrication of magnetotactic hydrogel container

A solution containing 50% (v/v) poly(ethylene glycol) diacrylate (*M*_n_: 700), 49.4% aqueous P123 solution (8 g l^−1^), and 0.6% 2-hydroxy-2-methylpropiophenone was prepared 30 min before the experiment. Rhombic rod foldectures **F1** (2.0 mg) were homogeneously suspended in prepolymer solution (30 μl, ∼1.1 × 10^7^ foldectures per μl) by vigorous agitation. The resulting mixture was gently poured into a PDMS mould, and the mould was placed between two Nd–Fe–B magnets for 2 min to achieve intact alignment of the foldectures before polymerization. While the magnetic field was applied, the foldecture–prepolymer suspension in the PDMS mould was photopolymerized under irradiation with 365-nm ultraviolet light (4 W, Vilber Lourmat, France) at room temperature. After irradiation for 40 min, a stiff hydrogel container enveloping the aligned foldectures was successfully obtained (Supplementary Fig. 10).

## Additional information

**How to cite this article:** Kwon, S. *et al*. Magnetotactic molecular architectures from self-assembly of β-peptide foldamers. *Nat. Commun.* 6:8747 doi: 10.1038/ncomms9747 (2015).

## Supplementary Material

Supplementary Information Supplementary Figures 1-10 and Supplementary Tables 1-2

Supplementary Movie 1 Orientational motion of the foldectures F1 suspended in water under rotating magnetic field. Schematic depiction of a magnetic compass indicates the direction of applied magnetic field. This movie corresponds to Figure 5a.

Supplementary Movie 2 Orientational motion of the foldectures F2 suspended in water under rotating magnetic field. Schematic depiction of a magnetic compass indicates the direction of applied magnetic field. This movie corresponds to Supplementary Fig. 9.

Supplementary Movie 3 Rhombus-shaped hydrogel container shows a magnetotactic behaviour, indicating the direction of applied magnetic field controlled by the laboratory-built rotating magnetic field device. This movie corresponds to Figure 5d and Supplementary Fig. 10.

## Figures and Tables

**Table 1:** Calculated diamagnetic susceptibilities along the orthorhombic crystallographic axes of foldectures **F1** and **F2**.

Cell axes	Diamagnetic susceptibility (10^−6^ cm^3^ mol^−1^)	
	Foldecture F1	Foldecture F2
*χ*_*a*_	−1,887.0	−2,683.7
*χ*_*b*_	−1,842.2	−2,640.1
*χ*_*c*_	−1,837.7	−2,713.7
