# Supplementary material for: Magnetotactic molecular architectures from self-assembly of β-peptide foldamers
Source: Nat Commun. 2015 Oct 29;6:8747. doi: 10.1038/ncomms9747 (PMC4640081; doi:10.1038/ncomms9747)
Supplement: Supplementary Information — Supplementary Figures 1-10 and Supplementary Tables 1-2 [file ncomms9747-s1.pdf]

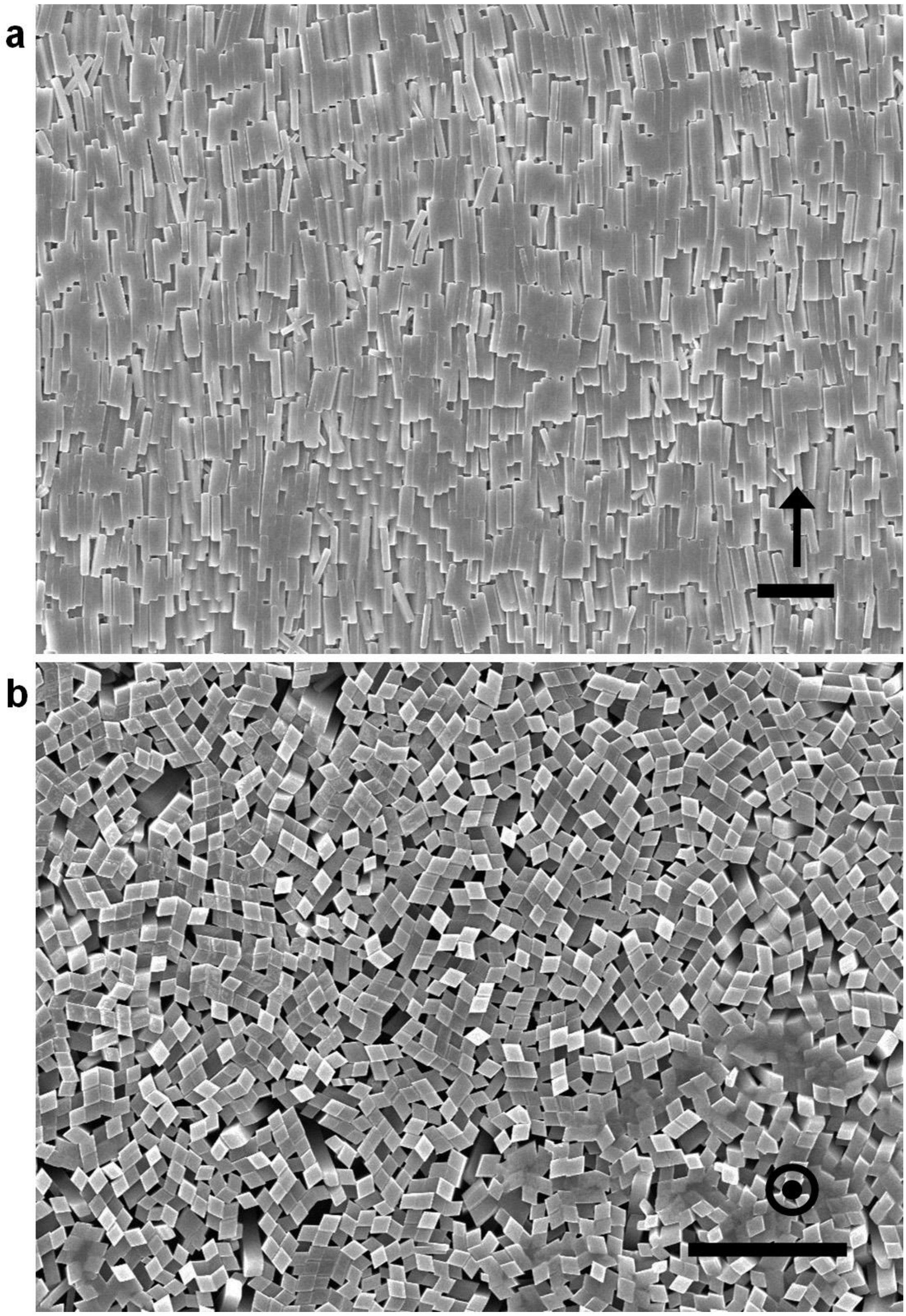


**Supplementary Figure 1.** Large-area SEM images of rhombic rod foldectures (**F1**) deposited on Si substrate in (**a**) an in-plane magnetic field and (**b**) an out-of-plane magnetic field. Scale bars: 10 μm.

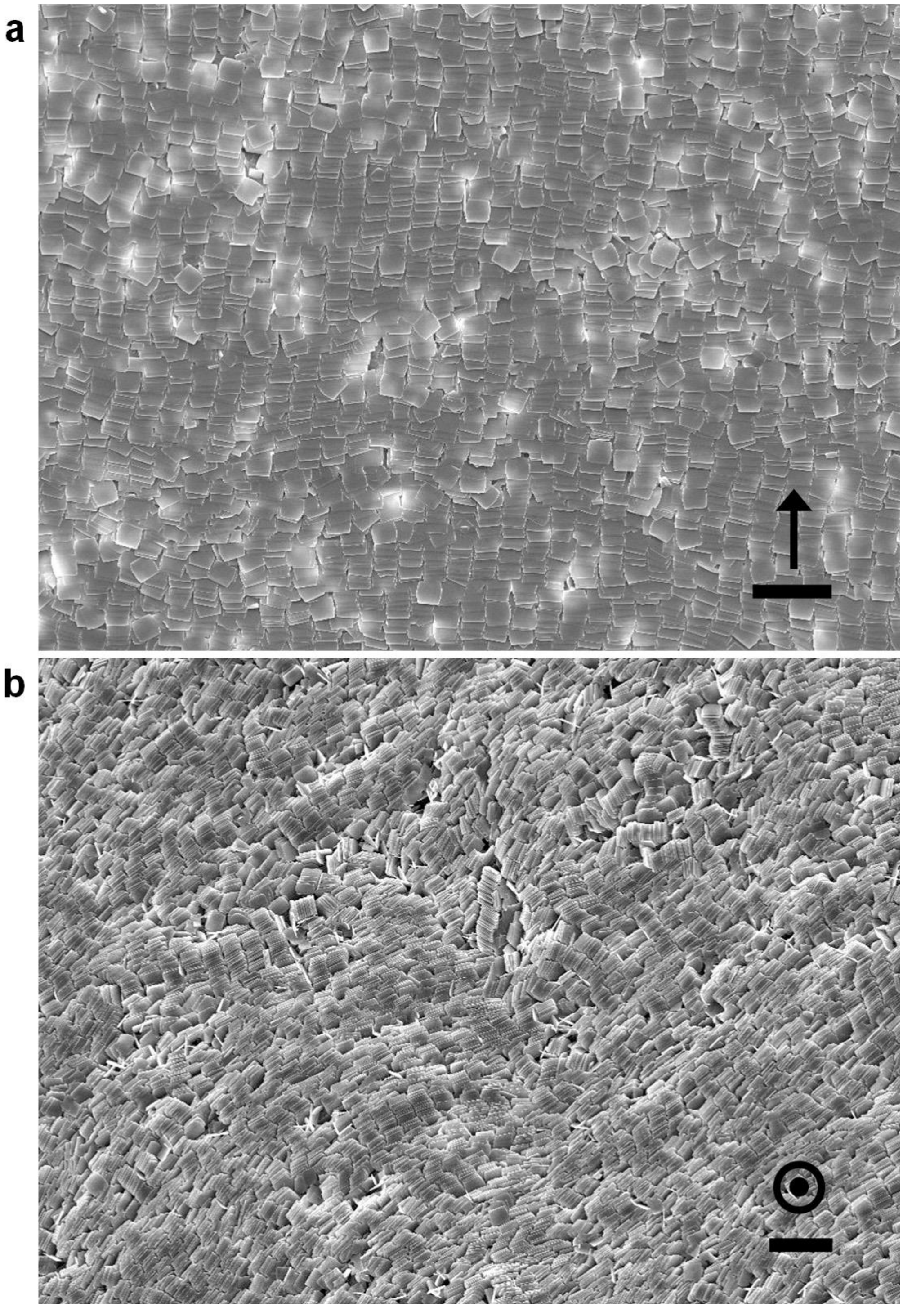


**Supplementary Figure 2.** Large-area SEM images of rectangular plate foldectures (**F2**) deposited on Si substrate in (**a**) an in-plane magnetic field and (**b**) an out-of-plane magnetic field. Scale bars: 10 μm.

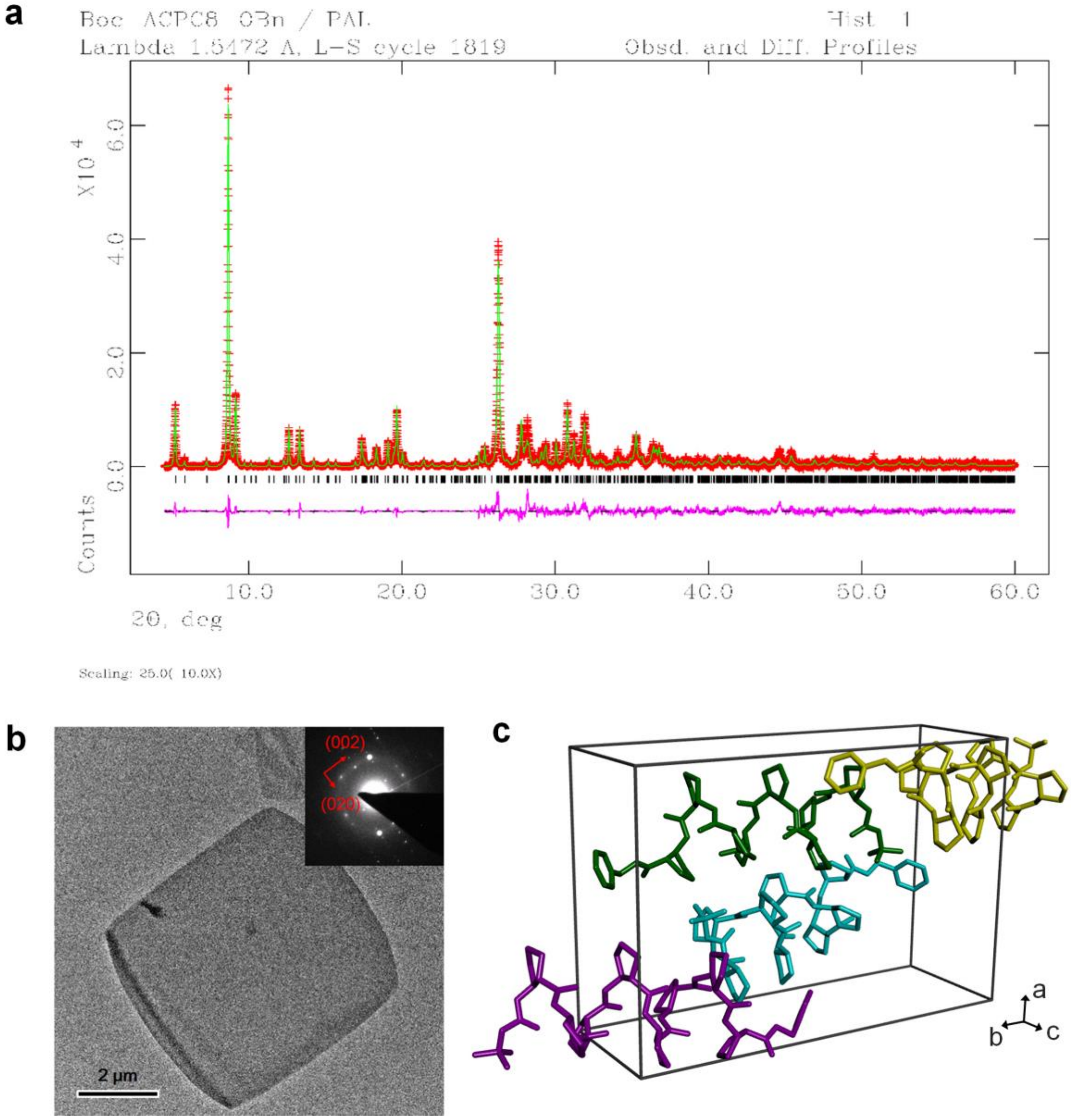


**Supplementary Figure 3.** (**a**) The final Rietveld plot for foldectures **F2**. The observed (red cross) intensities were superimposed on the calculated (green line) intensities. The difference profile was plotted as magenta line. The $2\theta$ range higher than 25° was magnified 10 times for clarity of high-angle data. (**b**) Transmission electron microscopy image of a **F2** and (inset) corresponding selected area electron diffraction pattern (from a view along [100] zone axis). Crystallographic *c* and *b* axes lie along the major and minor axes of the rectangular plate, respectively. (**c**) Molecular structure of foldecture **F2** in the unit cell.

**a**

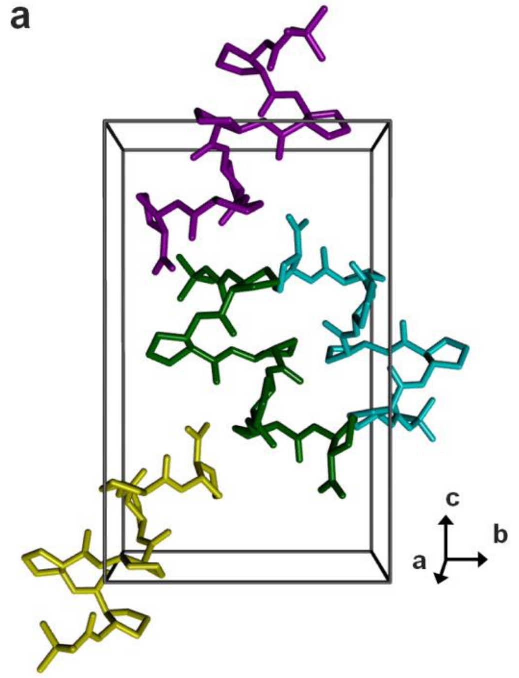


$\chi_{ij}$ ($10^{-6}$ cm$^3$ mol$^{-1}$)

| | | | |
|---|---|---|---|
| Peptide 1a | -471.8 | -3.4 | -1.1 |
| | -4.1 | -460.6 | 5.7 |
| | 1.2 | 5.0 | -459.4 |
| Peptide 1b | -471.7 | 3.4 | -1.1 |
| | 4.1 | -460.6 | -5.7 |
| | 1.2 | -5.0 | -459.4 |
| Peptide 1c | -471.7 | 3.4 | 1.1 |
| | 4.2 | -460.5 | 5.7 |
| | -1.2 | 5.0 | -459.4 |
| Peptide 1d | -471.8 | -3.4 | 1.1 |
| | -4.1 | -460.6 | -5.7 |
| | -1.2 | -5.0 | -459.4 |
| Total | -1887.0 | 0.0 | 0.0 |
| | 0.0 | -1842.2 | 0.0 |
| | 0.0 | 0.0 | -1837.7 |

**b**

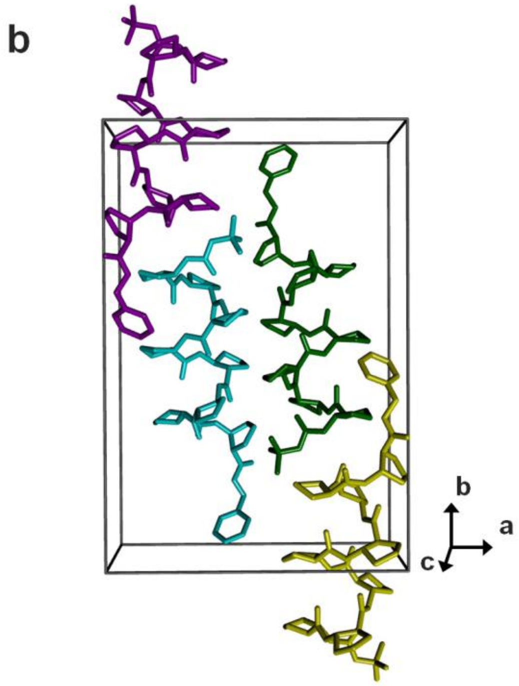


$\chi_{ij}$ ($10^{-6}$ cm$^3$ mol$^{-1}$)

| | | | |
|---|---|---|---|
| Peptide 2a | -670.9 | 12.0 | 13.2 |
| | 11.9 | -660.0 | -18.8 |
| | 12.9 | -19.3 | -678.4 |
| Peptide 2b | -670.9 | -12.0 | 13.2 |
| | -11.9 | -660.0 | 18.8 |
| | 12.9 | 19.2 | -678.4 |
| Peptide 2c | -670.9 | 12.0 | -13.2 |
| | 11.9 | -660.0 | 18.8 |
| | -12.9 | 19.3 | -678.4 |
| Peptide 2d | -670.9 | -12.0 | -13.2 |
| | -11.9 | -660.0 | -18.8 |
| | -12.9 | -19.3 | -678.5 |
| Total | -2683.7 | 0.0 | 0.0 |
| | 0.0 | -2640.1 | 0.0 |
| | 0.0 | 0.0 | -2713.7 |

**Supplementary Figure 4.** Calculated diamagnetic susceptibility tensors in crystal structures of (**a**) **F1** and (**b**) **F2**. Total diamagnetic susceptibility was estimated by linear combination of each susceptibility tensor for four peptide molecules, indicated by different colours (magenta, azure, green, and yellow).

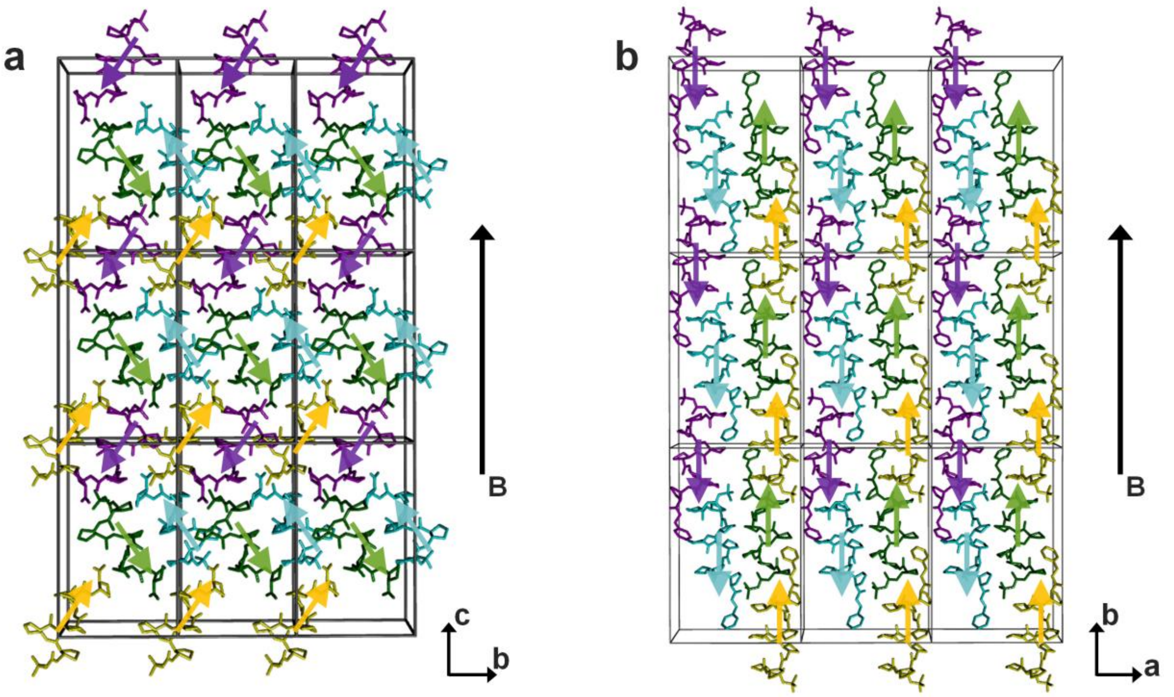


**Supplementary Figure 5.** Molecular packing structures of (**a**) **F1** and (**b**) **F2** from views along the *a*, *c* axes, respectively. Colored arrows indicate the direction of the helical axis (N termini to C termini).

**a**

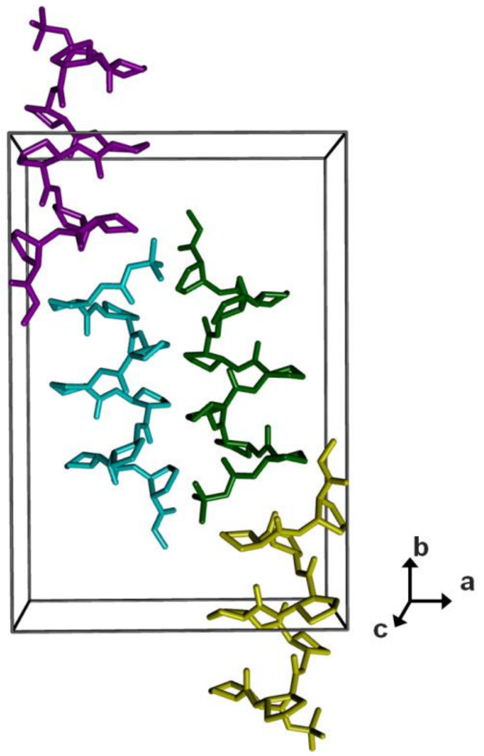


| | $\chi_{ij}$ ($10^{-6}$ cm$^3$ mol$^{-1}$) | | |
|---|---|---|---|
| Peptide 2a (methyl ester) | -630.6 | 0.6 | 2.1 |
| | 0.4 | -598.6 | 2.2 |
| | 2.0 | 1.8 | -623.6 |
| Peptide 2b (methyl ester) | -630.7 | -0.6 | 2.0 |
| | -0.4 | -598.6 | -2.2 |
| | 1.9 | -1.8 | -623.7 |
| Peptide 2c (methyl ester) | -630.7 | 0.5 | -2.1 |
| | 0.4 | -598.6 | -2.3 |
| | -2.0 | -1.8 | -623.7 |
| Peptide 2d (methyl ester) | -630.7 | -0.6 | -2.1 |
| | -0.4 | -598.6 | 2.2 |
| | -1.9 | 1.8 | -623.7 |
| Total | -2522.6 | 0.0 | 0.0 |
| | 0.0 | -2394.5 | 0.0 |
| | 0.0 | 0.0 | -2494.6 |

**b**

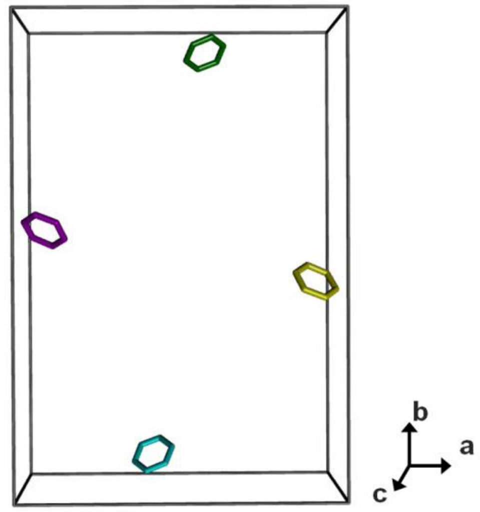


| | $\chi_{ij}$ ($10^{-6}$ cm$^3$ mol$^{-1}$) | | |
|---|---|---|---|
| Benzene a | -45.2 | 12.7 | 10.8 |
| | 12.8 | -66.2 | -22.6 |
| | 10.8 | -22.5 | -58.9 |
| Benzene b | -45.2 | -12.8 | 10.8 |
| | -12.8 | -66.2 | 22.6 |
| | 10.8 | 22.5 | -58.9 |
| Benzene c | -45.2 | 12.8 | -10.8 |
| | 12.8 | -66.2 | 22.6 |
| | -10.8 | 22.5 | -58.9 |
| Benzene d | -45.2 | -12.8 | -10.8 |
| | -12.8 | -66.2 | -22.6 |
| | -10.8 | -22.5 | -58.9 |
| Total | -180.7 | 0.0 | 0.0 |
| | 0.0 | -264.9 | 0.0 |
| | 0.0 | 0.0 | -235.6 |

**Supplementary Figure 6.** Contributions of (**a**) the peptides with methyl ester (BocNH-ACPC$_8$-OMe) and (**b**) C-terminal benzene groups to the total diamagnetic susceptibility in the crystal structure of **F2**.

**a**

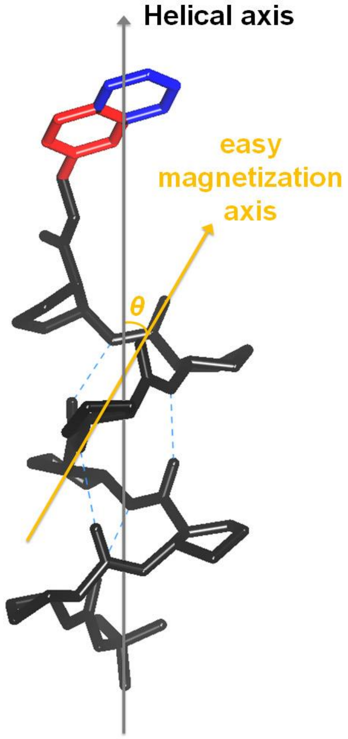


**b**

| | $\Delta\chi$ / $\theta$ (°) | | | |
|---|---|---|---|---|
| C-terminal Group | n = 6 | n = 8 | n = 10 | n = 12 |
| **Methyl** | 17.51 / 41.8 | 26.73 / 30.2 | 34.46 / 66.1 | 38.99 / 45.2 |
| **Benzyl** | 53.06 / 49.0 | 54.28 / 53.1 | 62.76 / 34.8 | 63.61 / 30.2 |
| **Naphthalen -2-ylmethyl** | 103.95 / 57.8 | 104.42 / 61.2 | 108.46 / 31.8 | 110.10 / 31.1 |

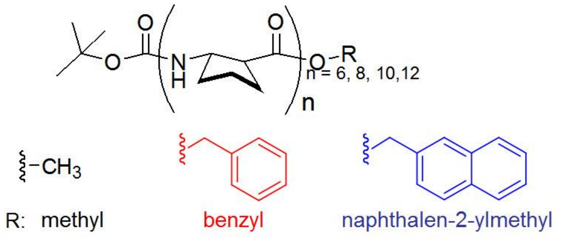


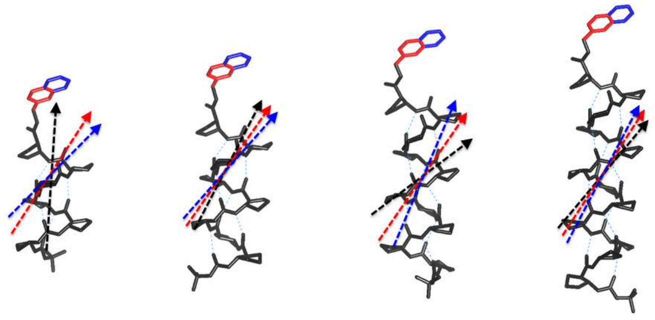


**Supplementary Figure 7.** (**a**) Angle ($\theta$) made from the helical axis (gray) of model peptides and easy magnetization axis (yellow) obtained from DFT calculation. (**b**) Table of the maximum diamagnetic anisotropic susceptibilities and estimated angles ($\theta$) for model 12-helical *trans*-ACPC homooligomers (BocNH-ACPC$_n$-OR, n = 6, 8, 10, 12) with various C-terminal functional groups (methyl, benzyl, and naphthalene-2-ylmethyl). Arrows indicate direction of the easy magnetization axis of each peptide (black: methyl, red: benzyl, blue: naphthalene-2-ylmethyl).

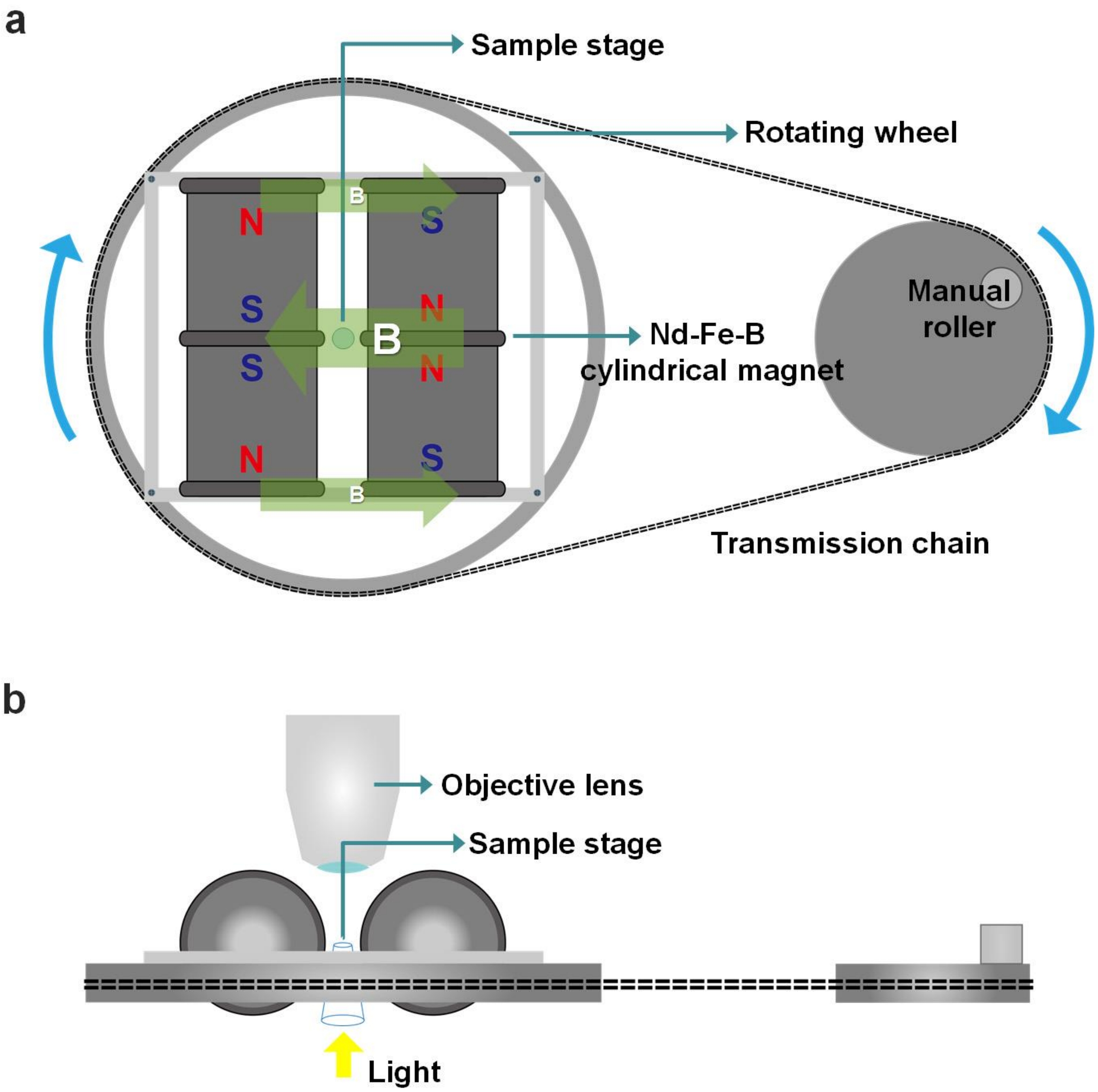


**Supplementary Figure 8.** (**a**) Top view and (**b**) side view of the experimental configuration for the optical microscope equipped with homebuilt rotating magnetic field device.

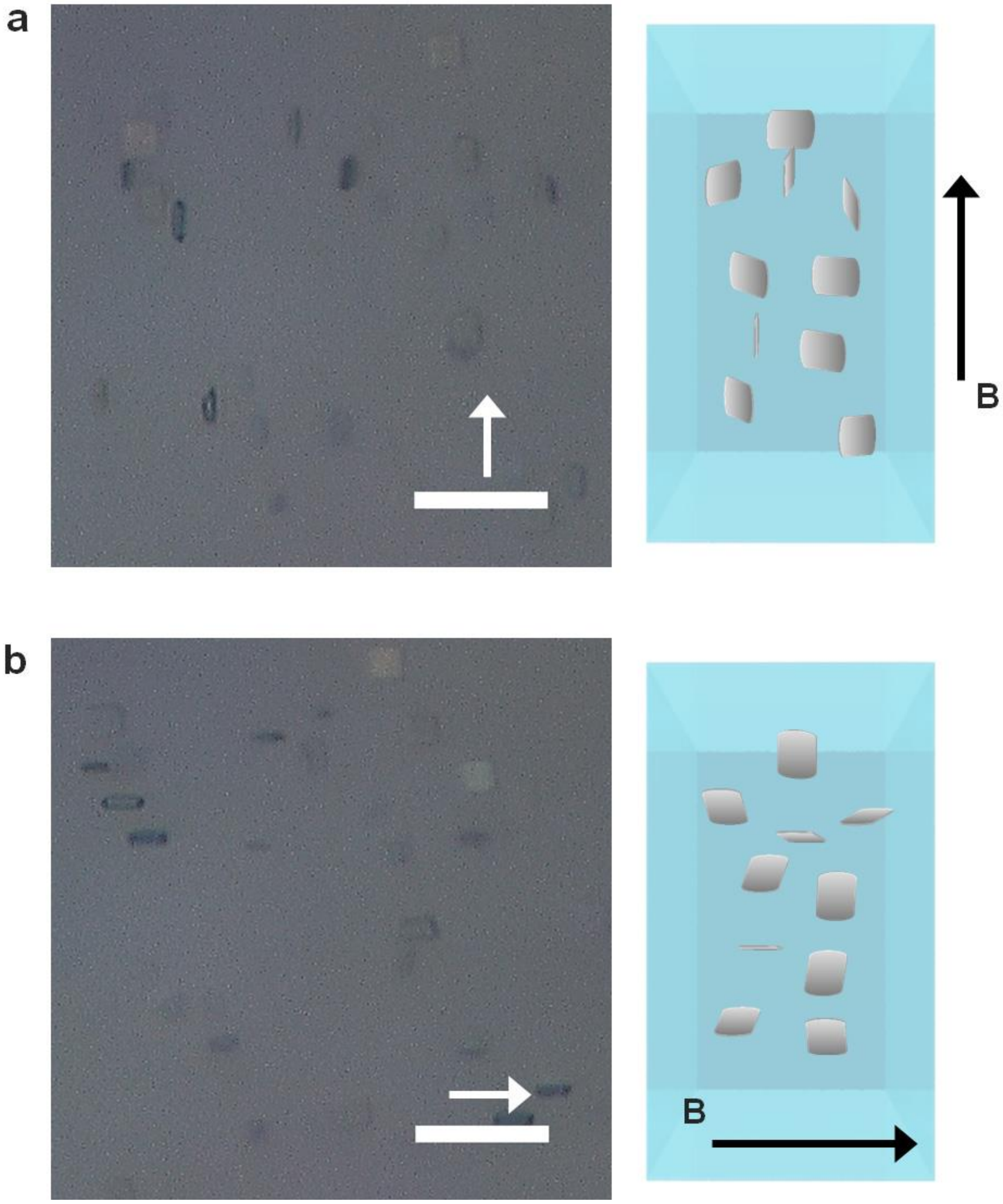


**Supplementary Figure 9.** (**a**,**b**) Snapshots taken from optical microscopy videos and schematic representations showing the foldectures **F2** suspended in water under rotating magnetic field. Arrows indicate the direction of magnetic field. Scale bars: 10 μm.

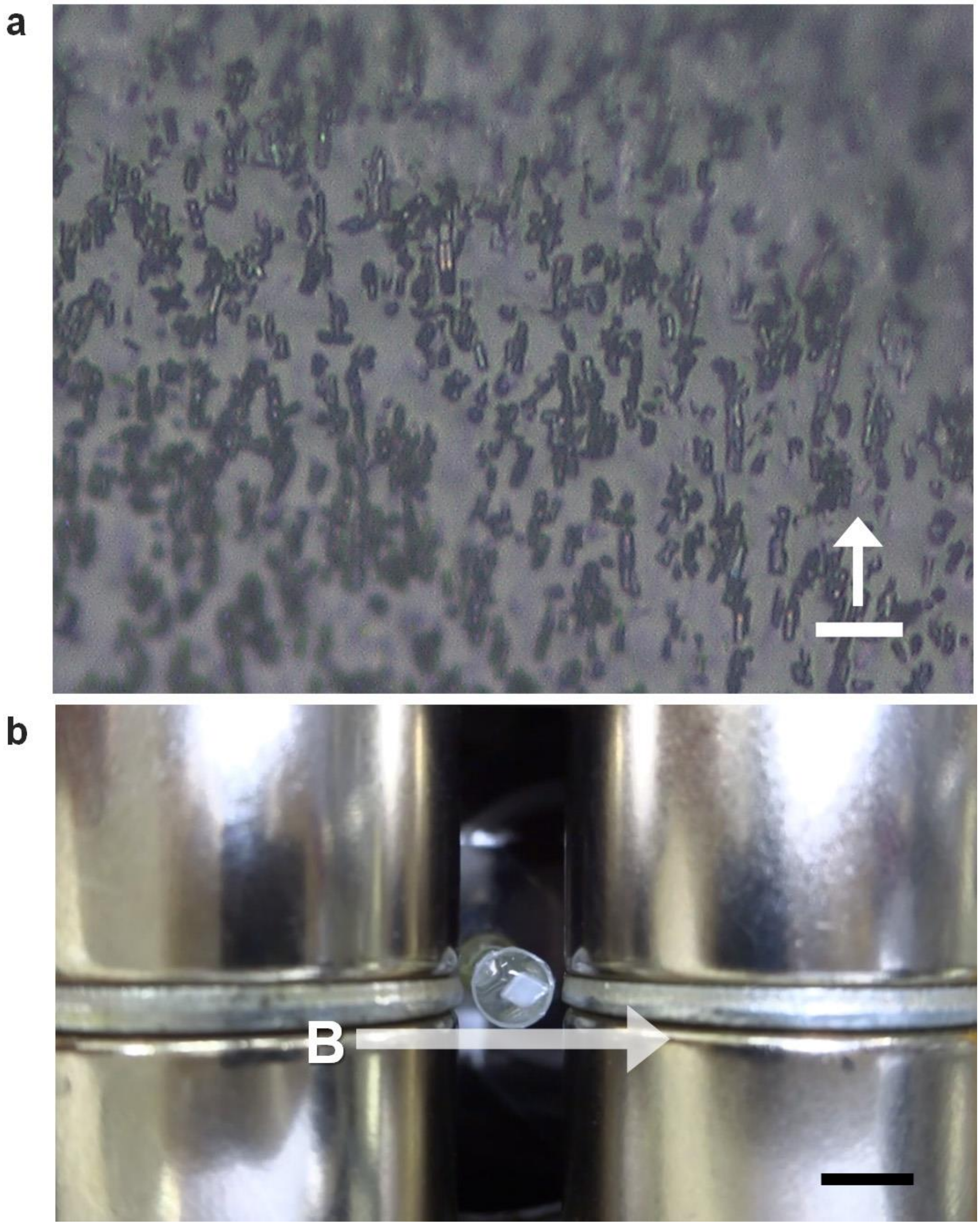


**Supplementary Figure 10.** (**a**) Optical microscopy image showing the aligned foldectures **F1** inside the hydrogel container. Scale bar: 10 μm. Arrow indicates the direction of applied magnetic field during photopolymerization process. (**b**) Photograph of rhombus-shaped macroscopic hydrogel container floating on the water with rotating magnetic field device. Horizontal magnetic field (**B**) is generated between the nodes of cylindrical magnets. Scale bar: 5 mm.

# Supplementary Tables

**Supplementary Table 1.** The powder diffraction experimental details of **F2**.

| | |
|---|---|
| Crystal data | |
| Chemical formula | $C_{60}N_8O_{11}$ |
| $M_r$ | 1008.70 |
| Cell setting, space group | Orthorhombic, $P2_12_12$ |
| Temperature (K) | Room temperature |
| *a*, *b*, *c* (Å) | 20.49696(14), 30.5198(5), 10.21122(18) |
| V(Å$^3$) | 6387.77(16) |
| Z | 4 |
| Radiation type | Synchrotron |
| Specimen form, color | Flat type (particle morphology: thin plate type powder) ,White |
| Specimen preparation temperature | Room temperature |
| | |
| Data Collection | |
| Diffractometer | 9B HRPD, PAL, Pohang, Republic of Korea |
| Data collection method | Specimen mounting: silicon non-reflection holder; mode: Reflection mode, Bragg-Brentano geometry; scan method: step, fixed θ |
| 2θ(°) | 2θmin =4.5, 2θmax=60, increment=0.005 |
| | |
| Refinement | |
| Refinement on | Observed intensities |
| Preferred orientation correction | March-Dollase model, for (100), $P_{MD}$=0.483200 |
| R Factors and goodness-of-fit | $R_p$=0.0597, $R_{wp}$=0.0772, $R_{exp}$=0.0332, S=2.33 |
| Wavelength of incident radiation (Å) | 1.5472 |
| Excluded region(s) | None |
| Profile function | Pseudo-voigt |
| No. of parameters | 240 |
| H-atom treatment | Omitted during the whole process for clarity |

**Supplementary Table 2.** Atomic coordination table of **F2**.

| Name | Atom type | x | y | Z | Occupancy | Uiso | Multiplicity |
|---|---|---|---|---|---|---|---|
| C1 | C | 0.4466(6) | 0.7513(6) | 0.3713(14) | 1 | 0.1097(7) | 4 |
| C2 | C | 0.4472(6) | 0.7171(5) | 0.5935(17) | 1 | 0.1097(7) | 4 |
| C3 | C | 0.4264(5) | 0.7973(5) | 0.5693(18) | 1 | 0.1097(7) | 4 |
| C4 | C | 0.41854(32) | 0.75263(27) | 0.5080(9) | 1 | 0.1097(7) | 4 |
| O5 | O | 0.34783(26) | 0.74399(24) | 0.4968(23) | 1 | 0.1097(7) | 4 |
| C6 | C | 0.32442(25) | 0.70288(19) | 0.4978(15) | 1 | 0.1097(7) | 4 |
| O7 | O | 0.35443(34) | 0.67046(31) | 0.4646(22) | 1 | 0.1097(7) | 4 |
| N8 | N | 0.26202(31) | 0.70299(25) | 0.5378(12) | 1 | 0.1097(7) | 4 |
| C9 | C | 0.21826(33) | 0.66634(28) | 0.5128(7) | 1 | 0.1097(7) | 4 |
| C10 | C | 0.1677(4) | 0.6599(7) | 0.6224(11) | 1 | 0.1097(7) | 4 |
| C11 | C | 0.1028(5) | 0.6707(9) | 0.5623(17) | 1 | 0.1097(7) | 4 |
| C12 | C | 0.1089(4) | 0.6575(6) | 0.4227(13) | 1 | 0.1097(7) | 4 |
| C13 | C | 0.17841(32) | 0.67167(26) | 0.3849(7) | 1 | 0.1097(7) | 4 |
| C14 | C | 0.2070(4) | 0.64471(17) | 0.2740(6) | 1 | 0.1097(7) | 4 |
| O15 | O | 0.2069(4) | 0.60445(29) | 0.2771(12) | 1 | 0.1097(7) | 4 |
| N16 | N | 0.2324(4) | 0.66752(26) | 0.1749(8) | 1 | 0.1097(7) | 4 |
| C17 | C | 0.26357(34) | 0.64706(29) | 0.0622(7) | 1 | 0.1097(7) | 4 |
| C18 | C | 0.2575(5) | 0.6746(5) | -0.0621(12) | 1 | 0.1097(7) | 4 |
| C19 | C | 0.3097(5) | 0.6567(9) | -0.1498(14) | 1 | 0.1097(7) | 4 |
| C20 | C | 0.3652(4) | 0.6414(6) | -0.0612(11) | 1 | 0.1097(7) | 4 |
| C21 | C | 0.33785(33) | 0.64100(25) | 0.0797(7) | 1 | 0.1097(7) | 4 |
| C22 | C | 0.3537(4) | 0.59919(17) | 0.1536(6) | 1 | 0.1097(7) | 4 |
| O23 | O | 0.3451(4) | 0.56294(30) | 0.1041(11) | 1 | 0.1097(7) | 4 |
| N24 | N | 0.3793(4) | 0.60452(23) | 0.2728(9) | 1 | 0.1097(7) | 4 |
| C25 | C | 0.40153(32) | 0.56811(27) | 0.3536(8) | 1 | 0.1097(7) | 4 |
| C26 | C | 0.4664(4) | 0.5778(6) | 0.4235(13) | 1 | 0.1097(7) | 4 |
| C27 | C | 0.4502(5) | 0.5806(7) | 0.5668(15) | 1 | 0.1097(7) | 4 |
| C28 | C | 0.3956(4) | 0.5492(5) | 0.5855(11) | 1 | 0.1097(7) | 4 |
| C29 | C | 0.35271(32) | 0.55567(24) | 0.4634(8) | 1 | 0.1097(7) | 4 |
| C30 | C | 0.31359(27) | 0.51517(17) | 0.4280(11) | 1 | 0.1097(7) | 4 |
| O31 | O | 0.33516(35) | 0.47812(29) | 0.4466(21) | 1 | 0.1097(7) | 4 |
| N32 | N | 0.25433(34) | 0.52247(24) | 0.3786(13) | 1 | 0.1097(7) | 4 |
| C33 | C | 0.21111(32) | 0.48727(28) | 0.3366(8) | 1 | 0.1097(7) | 4 |
| C34 | C | 0.1442(4) | 0.4887(7) | 0.4047(12) | 1 | 0.1097(7) | 4 |
| C35 | C | 0.0939(4) | 0.4926(8) | 0.2970(18) | 1 | 0.1097(7) | 4 |
| C36 | C | 0.1251(4) | 0.4734(5) | 0.1783(14) | 1 | 0.1097(7) | 4 |
| C37 | C | 0.19665(30) | 0.48806(26) | 0.1879(8) | 1 | 0.1097(7) | 4 |

| | | | | | | | |
|---|---|---|---|---|---|---|---|
| C38 | C | 0.24283(28) | 0.45835(18) | 0.1130(10) | 1 | 0.1097(7) | 4 |
| O39 | O | 0.2317(4) | 0.41903(29) | 0.1001(18) | 1 | 0.1097(7) | 4 |
| N40 | N | 0.29692(33) | 0.47728(25) | 0.0673(12) | 1 | 0.1097(7) | 4 |
| C41 | C | 0.35097(32) | 0.45238(28) | 0.0121(8) | 1 | 0.1097(7) | 4 |
| C42 | C | 0.3856(4) | 0.4766(4) | -0.0990(12) | 1 | 0.1097(7) | 4 |
| C43 | C | 0.4501(5) | 0.4541(9) | -0.1075(15) | 1 | 0.1097(7) | 4 |
| C44 | C | 0.4696(4) | 0.4441(6) | 0.0328(13) | 1 | 0.1097(7) | 4 |
| C45 | C | 0.40552(31) | 0.44259(25) | 0.1127(7) | 1 | 0.1097(7) | 4 |
| C46 | C | 0.3946(4) | 0.39903(18) | 0.1809(6) | 1 | 0.1097(7) | 4 |
| O47 | O | 0.4083(4) | 0.36414(30) | 0.1275(11) | 1 | 0.1097(7) | 4 |
| N48 | N | 0.3701(4) | 0.40124(23) | 0.3018(9) | 1 | 0.1097(7) | 4 |
| C49 | C | 0.3657(4) | 0.36342(25) | 0.3886(7) | 1 | 0.1097(7) | 4 |
| C50 | C | 0.3858(5) | 0.3741(4) | 0.5291(11) | 1 | 0.1097(7) | 4 |
| C51 | C | 0.3561(5) | 0.3377(6) | 0.6091(15) | 1 | 0.1097(7) | 4 |
| C52 | C | 0.2957(5) | 0.3217(4) | 0.5357(11) | 1 | 0.1097(7) | 4 |
| C53 | C | 0.2957(4) | 0.34542(24) | 0.4022(7) | 1 | 0.1097(7) | 4 |
| C54 | C | 0.2779(4) | 0.31559(18) | 0.2889(6) | 1 | 0.1097(7) | 4 |
| O55 | O | 0.2970(4) | 0.27745(31) | 0.2843(12) | 1 | 0.1097(7) | 4 |
| N56 | N | 0.2377(4) | 0.33271(26) | 0.1997(8) | 1 | 0.1097(7) | 4 |
| C57 | C | 0.21345(33) | 0.30829(31) | 0.0872(7) | 1 | 0.1097(7) | 4 |
| C58 | C | 0.1391(4) | 0.3108(7) | 0.0737(13) | 1 | 0.1097(7) | 4 |
| C59 | C | 0.1272(5) | 0.3034(8) | -0.0684(16) | 1 | 0.1097(7) | 4 |
| C60 | C | 0.1805(4) | 0.3284(6) | -0.1363(11) | 1 | 0.1097(7) | 4 |
| C61 | C | 0.24034(31) | 0.32549(29) | -0.0450(7) | 1 | 0.1097(7) | 4 |
| C62 | C | 0.29385(26) | 0.29610(23) | -0.0981(7) | 1 | 0.1097(7) | 4 |
| O63 | O | 0.2828(4) | 0.2687(4) | -0.1836(12) | 1 | 0.1097(7) | 4 |
| N64 | N | 0.35315(31) | 0.30246(28) | -0.0476(11) | 1 | 0.1097(7) | 4 |
| C65 | C | 0.40889(31) | 0.27487(25) | -0.0797(9) | 1 | 0.1097(7) | 4 |
| C66 | C | 0.4739(4) | 0.2994(4) | -0.0727(15) | 1 | 0.1097(7) | 4 |
| C67 | C | 0.4956(6) | 0.2949(6) | 0.0680(16) | 1 | 0.1097(7) | 4 |
| C68 | C | 0.4618(5) | 0.2545(5) | 0.1249(11) | 1 | 0.1097(7) | 4 |
| C69 | C | 0.4177(4) | 0.23615(24) | 0.0155(8) | 1 | 0.1097(7) | 4 |
| C70 | C | 0.44725(33) | 0.19684(19) | -0.0510(7) | 1 | 0.1097(7) | 4 |
| O71 | O | 0.4861(4) | 0.1985(4) | -0.1386(11) | 1 | 0.1097(7) | 4 |
| O72 | O | 0.42047(30) | 0.15989(25) | -0.0082(12) | 1 | 0.1097(7) | 4 |
| C73 | C | 0.4572(4) | 0.12004(29) | -0.0320(11) | 1 | 0.1097(7) | 4 |
| C74 | C | 0.43419(20) | 0.08500(24) | 0.0600(5) | 1 | 0.1097(7) | 4 |
| C75 | C | 0.37129(19) | 0.06865(26) | 0.0514(4) | 1 | 0.1097(7) | 4 |
| C76 | C | 0.35019(27) | 0.03594(25) | 0.1350(8) | 1 | 0.1097(7) | 4 |

| | | | | | | | |
|---|---|---|---|---|---|---|---|
| C77 | C | 0.39044(30) | 0.02089(21) | 0.2331(7) | 1 | 0.1097(7) | 4 |
| C78 | C | 0.45182(29) | 0.03864(15) | 0.2473(6) | 1 | 0.1097(7) | 4 |
| C79 | C | 0.47390(29) | 0.07001(27) | 0.1601(7) | 1 | 0.1097(7) | 4 |
